# Supplementary material for: End Sequence Analysis Toolkit (ESAT) expands the extractable information from single-cell RNA-seq data
Source: Genome Res. 2016 Oct;26(10):1397–410. doi: 10.1101/gr.207902.116 (PMC5052061; doi:10.1101/gr.207902.116)
Supplement: Supplemental Material [file supp_gr.207902.116_Supp_Materials.tar.gz › ESAT-esat_v0.1/doc/html/classumms_1_1core_1_1model_1_1score_1_1_window_score_1_1_abstract_window_score.html]

ESAT: umms.core.model.score.WindowScore.AbstractWindowScore Class Reference


ESAT


- Main Page
- Related Pages
- Packages
- Classes
- Files

- Class List
- Class Index
- Class Hierarchy
- Class Members

All Classes Namespaces Files Functions Variables Enumerator Pages

- umms
- core
- model
- score
- WindowScore
- AbstractWindowScore

Public Member Functions |
Protected Attributes |
List of all members

umms.core.model.score.WindowScore.AbstractWindowScore Class Referenceabstract

Inheritance diagram for umms.core.model.score.WindowScore.AbstractWindowScore:

[legend]

Collaboration diagram for umms.core.model.score.WindowScore.AbstractWindowScore:

[legend]

|  |  |
| --- | --- |
| Public Member Functions | |
|  | AbstractWindowScore (Annotation t) |
|  | |
| Annotation | getAnnotation () |
|  | |
| Public Member Functions inherited from umms.core.model.score.WindowScore | |
| double | getScore () |
|  | |

|  |  |
| --- | --- |
| Protected Attributes | |
| Annotation | annotation |
|  | |

## Constructor & Destructor Documentation

|  |  |  |  |  |  |
| --- | --- | --- | --- | --- | --- |
| umms.core.model.score.WindowScore.AbstractWindowScore.AbstractWindowScore | ( | Annotation | *t* | ) |  |

## Member Function Documentation

|  |  |  |  |  |
| --- | --- | --- | --- | --- |
| Annotation umms.core.model.score.WindowScore.AbstractWindowScore.getAnnotation | ( |  | ) |  |

Returns the underlying window

Implements umms.core.model.score.WindowScore.

Here is the caller graph for this function:

## Member Data Documentation

Annotation umms.core.model.score.WindowScore.AbstractWindowScore.annotation

protected

---

The documentation for this class was generated from the following file:

- java/umms/core/model/score/WindowScore.java


---

Generated on Fri Aug 15 2014 15:11:14 for ESAT by  

 1.8.7
